# Seedling and field assessment of wheat (*Triticum aestivum* L.) dwarfing genes and their influence on root traits in multiple genetic backgrounds

**DOI:** 10.1093/jxb/erac306

**Published:** 2022-07-08

**Authors:** Cathrine H Ingvordsen, Pieter-Willem Hendriks, David J Smith, Kathryn M Bechaz, Greg J Rebetzke

**Affiliations:** CSIRO, Agriculture and Food, Canberra ACTAustralia; CSIRO, Agriculture and Food, Canberra ACTAustralia; Charles Sturt University, School of Agriculture and Wine Sciences, Wagga-Wagga NSWAustralia; CSIRO, Agriculture and Food, Yanco NSW, Australia; New South Wales, Department of Primary Industries, Yanco NSW, Australia; CSIRO, Agriculture and Food, Canberra ACTAustralia; Lancaster University, UK

**Keywords:** Alternative dwarfing genes, breeding, drought, maternal, root architecture, seed size

## Abstract

Deployment of the *Rht-B1b and Rht-D1b* dwarfing genes helped facilitate the Green Revolution to increase wheat yields globally. Much is known of the influence of these genes on plant height and agronomic performance, but not of their effects on root architecture. We assessed 29 near-isogenic lines (NILs) representing 11 Green Revolution and alternative dwarfing genes across multiple genetic backgrounds for root architecture characteristics in controlled and field environments. Genetic background did not influence plant height, but had a small and significant (*P*<0.05) effect on root architecture. All dwarfing gene NILs were significantly (*P*<0.01) shorter compared with tall controls. The Green Revolution *Rht-B1b* and *Rht-D1b* sometimes had longer seedling roots but were not different from their respective tall controls for root depth in the field. The *Rht8*, *Rht12*, and *Rht18* dwarfing gene NILs produced long seminal roots in seedling pouches, and a greater maximum rooting depth (MRD) and root penetration rate (RPR) in the field. Genotypic increases in MRD and RPR were strongly correlated with increased harvest index and grain yield, particularly in dry environments. Careful root phenotyping highlights the potential of novel dwarfing genes for wheat genetic improvement under water-limited conditions.

## Teaser

Dwarfing genes assessed across multiple genetic backgrounds varied in their influence on root architecture in seedling and adult wheat plants to increase harvest index and grain yield under drought.

## Introduction

Plant height of wheat was significantly reduced with the introduction of reduced height (*Rht*) genes distributed globally with the Green Revolution. The Green Revolution represented a scientific-led framework to increase global grain production and food security through integration across multidisciplinary research activities. Improved management strategies for irrigation and fertilization were coordinated with deployment of high-yielding germplasm with improved resource utilization efficiency supported by greater investment in crop research, and improved infrastructure and policy support (Pingali, 2012). The linking of high-yielding germplasm, photoperiod insensitivity, and major dwarfing genes in varietal development has been estimated to have increased wheat productivity ~1.0% per year (Evenson and Gollin, 2003; Lumpkin, 2015).

The most widely used dwarfing genes in wheat breeding are *Rht-B1b* (formerly *Rht1*) and *Rht-D1b* (formerly *Rht2*) bred from the Japanese wheat landrace Daruma. Both dwarfing genes reduce plant height through reduced sensitivity to the growth hormone gibberellic acid (GA). This GA insensitivity reduces cell expansion to decrease cell length and leaf and stem size (Keyes *et al.*, 1989; Peng *et al.*, 1999). Another group of dwarfing genes, termed ‘alternative dwarfing genes’, reduce plant height while maintaining GA sensitivity (Rebetzke *et al.*, 2012). One GA-sensitive (GAS) dwarfing gene, *Rht8*, was derived from the Japanese wheat landrace Akakomugi, and reduces plant height through sensitivity to brassinosteroids (Gasperini *et al.*, 2012). This gene is associated with greater cell size in seedling tissue to increase leaf area and coleoptile length (Botwright *et al*., 2001, 2005).

The successful adoption of wheat dwarfing genes was evidenced by widespread adoption of *Rht-B1b*, *Rht-D1b*, or *Rht8* in >70% of wheat varieties grown globally by the 1990s (Evans, 1998). Several studies have evaluated the agronomic and physiological performance of the Green Revolution dwarfing genes (e.g. Flintham *et al.*, 1997; Casebow *et al.*, 2016). Shorter elongating stems reduce competition to increase assimilate partitioning to ears, thereby increasing floret fertility and kernel number, and ultimately harvest index (HI) (Fischer and Stockman, 1986; Flintham *et al.*, 1997; Butler *et al.*, 2005). Both *Rht-B1b* and *Rht-D1b* are molecularly and physiologically similar, reducing plant height by 14–25% depending on genetic background and environment (Flintham *et al.*, 1997). In contrast, the alternative *Rht8* dwarfing gene, present in many eastern European varieties due to chromosomal linkage with the photoperiod-insensitive *Ppd-D1a* gene, is more variable in its plant height reduction (8–38%) (Ellis *et al.*, 2004; Rebetzke *et al.*, 2012).

Despite their widespread adoption, several studies have reported a negative influence of the GA-insensitive (GAI) dwarfing genes on agronomic performance particularly in less favourable growing environments. For example, Butler *et al.* (2005) reported that non-*Rht* tall lines were equal to, or greater than, GAI *Rht-B1b* and *Rht-D1b* siblings in grain yield, test weight, and kernel weight across contrasting irrigation regimes. Similar reductions in biomass and yield have also been reported elsewhere for *Rht-B1b* and *Rht-D1b*, and particularly when assessed in droughted environments (e.g. Mathews *et al.*, 2006; Jatayev *et al.*, 2020). The GAI dwarfing genes have been widely reported to reduce coleoptile length to reduce seedling establishment especially when sown deep (Trethowan *et al.*, 2001; Ellis *et al.*, 2004; Rebetzke *et al.*, 2007). Further, *Rht-B1b* has been reported to reduce photosynthetic rate in the flag leaf and reduce grain protein content (Jobson *et al.*, 2019).

All dwarfing genes have been reported to decrease canopy height, but their influence on root growth and architecture are not well understood. Comparisons between dwarfing genes have largely been restricted to seedling root architecture in controlled environments (e.g. McCaig and Morgan, 1993; Wojciechowski *et al.*, 2009) with some field assessment of rooting depth and root length density (e.g. Siddique *et al.*, 1990; Hodgkinson *et al.*, 2017). Genetic assessment has focused on mapping populations (e.g. Bai *et al.*, 2013), comparisons between historic and modern wheat varieties (e.g. Waines and Ehdaie, 2007; Friedli *et al.*, 2019), and less frequently between dwarfing gene near-isogenic lines (NILs) (e.g. Miralles *et al.*, 1997; Wojciechowski *et al.*, 2009). The wide range of experimental and sampling methods, and inconsistency in findings between studies, has highlighted uncertainty in the influence of wheat dwarfing genes on root growth (e.g. McCaig and Morgan, 1993; Miralles *et al.*, 1997).

Deeper roots are hypothesized to increase late-season access to water and nutrients particularly in rainfed environments. Kirkegaard *et al.* (2007) demonstrated how uptake of even small amounts (10.5 mm) of water from depth later in the season increased water use efficiency to significantly increase grain yield in rainfed wheat crops. Deeper roots have also been suggested as a key breeding objective for accessing additional nutrient and water resources from greater soil depth to increase wheat yields (Araus *et al.*, 2002; Wasson *et al.*, 2014). However, despite the well-established influence of dwarfing genes on shoot growth (e.g. Botwright *et al.*, 2005; Rebetzke *et al.*, 2014), how dwarfing genes influence root growth in wheat is poorly understood. In this study, we assessed root growth for 11 dwarfing genes representing multiple genetic backgrounds in seedling and adult plants in controlled and field environments.

## Materials and methods

### Wheat genotypes

Wheat genotypes were contained in four sets representing different wheat genetic backgrounds and containing between 2 and 11 different dwarfing (*Rht*) genes (Tables 1–3). Set one contained CSIRO-developed, BC_5-6_-derived dwarfing gene NILs in the tall, Australian variety ‘Halberd’. A representative tall sister NIL (‘HalberdT’) was selected and used as the tall control for direct comparisons with each of the dwarfing gene NILs containing either *Rht-B1b*, *Rht-D1b*, *Rht-B1c*, *Rht-D1c*, *Rht5*, *Rht8*, *Rht9*, *Rht12*, *Rht13*, or *Rht18*. Seven lines were randomly sampled for each dwarfing gene to minimize bias through sampling of genetic background. Set two contained BC_4_-derived NILs for six dwarfing genes (*Rht-B1b*, *Rht-D1b*, *Rht-B1c*, *Rht4*, *Rht5*, and *Rht8*) and the tall parent in the Russian ‘spring’ variety Miranovskaya (hereafter ‘M808S’) (Loskutova, 1998). Set three consisted of four CIMMYT-based, spring maturity pairs near isogenic for *Rht-B1b* (Galvez 87, Nesser 90, and Seri 82) and *Rht-D1b* (Pavon 76), and their tall siblings (Trethowan *et al.*, 2001). All NILs were BC_5_ derived. Set four contained CSIRO-developed, BC_3_-derived *Rht13* or *Rht18* NILs and their *Rht-B1b* or *Rht-D1b* parent, and represented six modern Australian wheat varieties: Espada, Gregory, Mace, Magenta, Scout, and Yitpi. Together, the study contained NILs representing four GAI (*Rht-B1b*, *Rht-D1b*, *Rht-B1c*, and *Rht-D1c*) and seven GAS (*Rht4*, *Rht5*, *Rht8*, *Rht9*, *Rht12*, *Rht13*, and *Rht18*) dwarfing genes, and tall height controls.

### Seedling root screen in a controlled environment

All seeds were harvested from plants grown in the same well-managed glasshouse environment before carefully hand-threshing and storing air-dry at room temperature. Seeds were inspected to be free of any visible damage and then standardized to a common weight of 47 (±0.5) mg. At sowing, weighed seed were soaked in Thiram® fungicide (1.4 g l^–1^) for 3 min and two seeds of each genotype were then positioned between two 25.5 × 38.1 cm sheets of germination paper (Anchor Premium®) to make a ‘pouch’ around each seed. Seeds were placed embryo down, 85 mm apart, before fixing to a steel rod with plastic-covered paperclips. The pouch was then suspended vertically in a custom-made, black Perspex box to exclude light from the growing roots. Water was added daily to the base of each box to keep sheets moist. Seed were stratified after sowing in the dark at 4 °C for 3 d, and boxes then moved to a growth chamber with a 12 h day/night cycle, light intensity of 600 μmol m^−2^ s^−1^, and air temperatures of 20/15 °C (day/night). The experiment was terminated after 12 d when the first roots reached the bottom of the box.

At harvest, measurements were made of the lengths of leaves one and two, and then entire shoots were dried at 72 °C for 3–4 d before weighing. Numbers and lengths of seminal roots and any branch roots were recorded before drying and weighing as for shoots. The angles between seminal roots two and three, and four and five were determined using a protractor with roots laid flat against the germination sheet. Length of roots was determined digitally with a custom-made program script using Fiji version 2.1.0 (Schindelin *et al.*, 2012) and applied to RGB photos taken at 5184 × 3456 pixel resolution under UV light in a custom-made box (modified after Wasson *et al.*, 2016).

### Field experiment management

A series of experiments were conducted in 2017 and 2018 at two sites in the southern wheatbelt of New South Wales, Australia: the Department of Primary Industries Research Station at Yanco (–34.628164S, 146.431761E; elevation 136 m); and the Neil Fettell Irrigation Centre at Condobolin (–33.047495S, 147.238776E; elevation 195 m). The Yanco experiments were undertaken at the Yanco Managed Environment Facility (see Rebetzke *et al.*, 2013 for details).

**Fig. 1.**
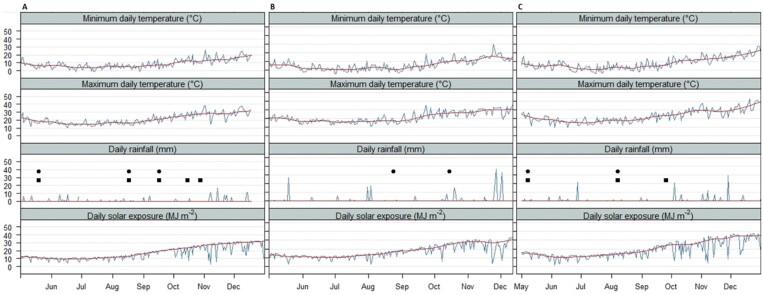
Weather conditions for Yanco 2018 (A), Condobolin 2017 (B), and Condobolin 2018 (C). The red line represents the smoothed weather curve, and actual measurements are the blue line. Irrigation events are shown for pre-anthesis irrigation (filled circles) and pre- and post-anthesis irrigation (filled squares) regimes. Average anthesis date was (A) 13 Oct (B) 7 Oct (C) 8 Oct.

Experiments at both sites were managed according to standard practice with plots sown mid- to late May following a pulse crop rotation and summer fallow. At Yanco, fertilizer was supplied as 100 kg ha^–1^ MAP (Incitec Pivot Fertilisers) with the seed while at Condobolin, fertilizer was supplied as 105 kg ha^–1^ Starter 15 (Incitec Pivot Fertilisers) also with the seed. Nitrogen requirements were monitored and supplemental urea (Incitec Pivot Fertilisers) was supplied as required based on crop growth predictions with the TopCrop® modelling program. Experiments were baseline rainfed, with two supplemental irrigation regimes being applied at each location (Fig. 1). In irrigation regime one (I1), irrigation was supplied up to anthesis (Z55–60) and then stopped (i.e. terminal drought with a final irrigation supplied at Z45–7). Irrigation regime two (I2) was as for I1 but with 1–2 additional irrigations at and after anthesis. Supplemental irrigation was supplied as needed to achieve: (i) the long-term average in-season rainfall with regime I1; and (ii) 25% above average in-season rainfall with regime I2. Timing and amounts of irrigation were determined using soil type and rainfall input data in the StressMaster® modelling program. At Yanco, irrigation was supplied by a travelling irrigator, and in 2018, a pre-sowing irrigation of 158 mm was applied. After sowing and during vegetative growth, a total of 112 mm of irrigation was supplied in five events in I1 and I2, and an additional 93 mm in I2 applied across three events post-anthesis (Fig. 1). At Condobolin, water was supplied with flood irrigation and the amount of water was estimated at 100 mm per irrigation event. The soil profile at Condobolin was full prior to sowing in 2017 so only a single irrigation was applied during vegetative growth (I1 and I2) and one irrigation at anthesis (I2). Irrigation at Condobolin in 2018 was as for 2017 except that an additional flood irrigation was supplied pre-sowing in both I1 and I2. The soil at Yanco is classified as a red chromosol (Merungle loam to Merungle sand) and Condobolin as a grey vertosol (Isbell, 1996).

At Yanco, all four sets of lines were grown under the two irrigation regimes (I1 and I2) in a p-rep (r=1.5) row–column experimental design with four blocks (two blocks per irrigation regime). Space was limited at Condobolin so only the Halberd NILs were grown in 2017 under I2 and in 2018 under both irrigation regimes (I1 and I2). Genotypes at Condobolin were replicated four times in a row–column experimental design. Irrigation treatments were applied as a split-plot design (irrigation as whole plot) at Condobolin and as a block design with irrigation levels randomized to blocks at Yanco.

In all experiments, 6 m long plots were sown at a target density of 160 plants m^–2^ using seed from irrigated field trials harvested the previous year. Plots at Yanco were seven rows wide and spaced 25 cm apart, and at Condobolin were 10 rows wide and spaced 18 cm apart. At maturity, plots were end-trimmed to 5 m length and border rows were removed, leaving the middle rows for harvest.

Weather data were obtained from on-site weather stations or a nearby weather station provided through the Australian Government Bureau of Meteorology weather service (www.bom.gov.au).

### Field measurements

Mature plant height and grain yield were recorded for each plot. Plant height was measured in the middle row as the distance from the ground to the top of the head excluding the awns. Grain yield (per unit area) was calculated from the weight of machine-harvested grain and measured plot length and width (as number of rows×spacing). At Yanco, anthesis date (Zadoks *et al.*, 1974), anthesis biomass, and HI were measured, and maturity biomass calculated. For anthesis biomass, four bordered rows of 30 cm were cut at ground level at anthesis (Z65), dried for 4 d at 72 °C, and weighed. For HI, three 50 cm long bordered rows were cut at maturity (Z99), weighed, and then threshed. The HI was calculated as threshed grain weight÷total bundle weight, and maturity biomass was calculated as plot yield÷HI.

Maximum root depth was determined for each plot after harvest using a modification of the core-break method (Wasson *et al.*, 2014). Briefly, 4 × 4.2cm diameter soil cores were inserted in the soil to a depth of 2 m using a tractor-mounted, hydraulic soil corer. Two cores were taken on, and two cores between, sowing rows in the middle of each plot. Intact cores were carefully placed into a cradle and the length of the soil core was noted. Each core was manually broken into 5 cm lengths from the bottom of the core, and the depth and numbers of visible roots were recorded for each of the broken surfaces. Trained scorers were allocated to cores at random. When roots were identified and counted at three consecutive depths (e.g. at 120, 115, and 110 cm), the core was discarded and maximum root depth (MRD) recorded (after Wasson *et al.*, 2014). A second derived measure, root penetration rate (RPR), was calculated for plots at Yanco as: MRD÷days from sowing to flowering (after Kirkegaard and Lilley, 2007).

### Statistical analysis

A combined analysis of variance and covariance over sites, years, and irrigation regimes was performed for all characters using the SAS mixed linear models procedure Proc MIXED (Littell *et al.*, 1996). Dwarfing gene, genetic background, and irrigation regime were assumed fixed effects, whilst blocks, sites, and years were random effects in the linear model containing both main effects and their interactions. The general statistical model was:


$$\begin{array}{l} \begin{array}{ll} Y_{ijklr}= & \;m+\;L_{i}+\;Y_{j}\\ & +\;LY_{ij}+R/LY_{r(ij)}\\ & +\;I_{k}+\;LI_{ik}+\;YI_{jk}+\;LYI_{ijk}\\ & +\;R/LYI_{r(ijk)}+\;G_{l}+\;GL_{li}\\ & +\;GY_{lj}+GI_{lk}+\;GLY_{lij}+\;GLI_{lik}+\;GYI_{ljk}\\ & +\;GLYI_{lijk}+\;e_{ijklr} \end{array} \end{array}$$

where m is the experiment mean, L_i_ is the effect of the ith location; Y_j_ is the effect of the jth year; LY_ij_ is the effect of the interaction between location i and year j; R/LY_r(ij)_ is the effect of the rth block nested in the ijth location and year; I_l_ is the effect of the lth irrigation; LI_ik_ is the effect of the interaction between location i and irrigation l; YI_jk_ is the effect of the interaction between year j and irrigation k; LYI_ijk_ is the effect of the interaction between location i, year j, and irrigation k; R/LYI_r(ijk)_ is the effect of the rth block nested in the ijkth location, year, and irrigation; G_l_ is the effect of the lth genotype; GL_lk_ is the effect of the interaction between genotype l and location I; GY_lj_ is the effect of the interaction between genotype l and year j; GI_lk_ is the effect of the interaction between genotype l and irrigation k; GLY_lij_ the effect of the interaction between genotype l, location I, and year j; GLI_lik_ is the effect of the interaction between genotype l, location I, and irrigation k; GYI_ljk_ is the effect of the interaction between genotype l, year j, and irrigation k; GLYI_lijk_ is the effect of the interaction between genotype l, location I, year j, and irrigation k; and e_ijklr_ is the residual.

Years, locations, and blocks were random effects, and irrigation and genotypes were fixed effects. The model was modified replacing genotype terms with genetic background and NILs nested within genetic backgrounds when comparing specific dwarfing gene effects. Further, appropriate site and year main and interaction effects were removed when analysis was undertaken for specific sites (e.g. Condobolin versus Yanco) and/or years (e.g. Yanco 2018 irrigated and rainfed).

In a separate analysis, genotypes were considered as random effects to obtain variance and covariance estimates in the linear mixed model. For root measurements, individual scorers undertaking root measures and the measured core length were included as fixed linear covariates in the mixed model. Genetic correlations and their SEs were estimated for all characters after Holland (2006). The testing of NIL means was undertaken using *a priori* pre-planned contrasts which use a 1 degree of freedom test under the null hypothesis of no dwarfing gene effect. In the mixed model, all fixed effects were tested with a Wald’s test. Heritability was estimated on an entry-mean basis after Holland *et al.* (2003). The effects of individual dwarfing genes on measured characters was determined in statistical comparisons with the relevant near-isogenic tall (Halberd, M808S, and CIMMYT) or *Rht-B1b/Rht-D1b* (Australian varietal) controls using an orthogonal linear vector fitted in the CONTRAST statement in Proc MIXED (Littell *et al.*, 1996). Unless otherwise indicated, statistical significance is reported at α=0.05. Figures were constructed in R version 3.5.2 (R Core Team, 2013) and SigmaPlot version 14.0. (Systat Software, San Jose, CA, USA).

## Results

### Seedling measurements in controlled environments

Seedlings were harvested at approximately the 1.6 leaf stage (Supplementary Table S1). In the Halberd genetic background, the length of the first seedling leaf was significantly (*P*<0.01) reduced in lines containing the GAI dwarfing genes (*Rht-B1b*, *Rht-D1b*, *Rht-B1c*, and *Rht-D1c*) compared with the tall Halberd control (Table 1). The GAS dwarfing gene NILs were not different in length from the tall controls. The effect of dwarfing genes was less evident on the length of the second leaf as it was not fully elongated (Supplementary Table S1). All NILs had decreased length of leaf one in the M808S background, with significant reductions for the *Rht-D1b*, *Rht-B1c*, and *Rht4* dwarfing gene NILs. In the Australian varietal backgrounds, length of leaf one in *Rht-B1b* or *Rht-D1b* parental NILs was commonly reduced or equal in size to the *Rht13* and *Rht18* NILs. The only exception was in the Yitpi background where the *Rht-D1b* parent was significantly (*P*<0.01) greater for length of leaf one (Table 1). Averaged across varietal backgrounds, *Rht13* (+11%) and *Rht18* (+7%) were significantly greater in length than their *Rht-B1b* and *Rht-D1b* parents.

**Table 1.** Seedling leaf and root means for dwarfing gene near-isogenic lines (NILs) representing multiple genetic backgrounds

**Background/NIL**	**Length leaf 1 (mm)**	**No. of seminal roots (*n*)**	**Total seedling root length (mm)**	**Total seminal root length (mm)**	**Average seminal root length (mm)**
*Halberd*					
HalberdT (Tall)	144.7	4.65	1357	1230	266
Halberd *Rht-B1b*	127.8**	5.56**	1450	1435*	258
Halberd *Rht-D1b*	125.8**	5.04*	1341	1341	266
Halberd *Rht-B1c*	97.8**	5.00*	1458	1368	274
Halberd *Rht-D1c*	68.9**	5.25*	1390	1264	240*
Halberd *Rht5*	137.9	4.92	1375	1311	267
Halberd *Rht8*	143.2	4.81	1384	1333	277
Halberd *Rht9*	135.4	4.56	1227	1227	269
Halberd *Rht12*	135.7	4.92	1544*	1411*	287
Halberd *Rht13*	155.6	4.89	1457	1351	276
Halberd *Rht18*	137.9	5.11*	1495	1365*	267
*M808S*					
M808S (Tall)	157.3	4.33	1200	1200	277
M808S *Rht-B1b*	142.0	4.67	1373	1373	294
M808S *Rht-D1b*	134.4*	5.20*	1466*	1466*	282
M808S *Rht-B1c*	99.7**	4.50	1287	1287	286
M808S *Rht4*	130.8*	4.00	1352	1282	321**
M808S *Rht5*	140.7	5.00	1275	1256	251
M808S *Rht8*	141.7	4.33	1183	1133	262
*Australian varietal backgrounds*					
cv. Espada (*Rht-D1b*)	119.2	4.60	1193	1148	250
Espada *Rht13*	125.8	4.67	1257	1257	269
Espada *Rht18*	118.5	3.83*	1040	1040	272*
cv. Gregory (*Rht-B1b*)	118.0	4.83	1327	1288	267
Gregory *Rht13*	138.5	4.83	1217	1191	247
Gregory *Rht18*	116.8	4.60	1257	1257	273
cv. Mace (*Rht-D1b*)	103.8	5.20	1231	1231	237
Mace *Rht18*	122.8*	5.20	1412	1412*	272*
cv. Magenta (*Rht-D1b*)	112.0	5.00	1540	1539	308
Magenta *Rht18*	140.4**	4.80	1332	1332	278
cv. Scout (*Rht-D1b*)	99.3	4.83	1008	1008	209
Scout *Rht18*	108.6	4.40	1213*	1134*	258**
cv. Yitpi (*Rht-D1b*)	128.0	5.40	1518	1495	277
Yitpi *Rht18*	112.8**	5.25	1301**	1288**	245

*Rht*, reduced major height gene with *Rht-B1b*, *Rht-B1c*, *Rht-D1b*, and *Rht-D1c* genes conferring GA insensitivity (GAI), and *Rht4*, *Rht5*, *Rht8, Rht9*, *Rht12*, *Rht13*, and *Rht18* genes conferring GA sensitivity (GAS). Significance levels represent comparisons with the tall or *Rht-B1b*, *Rht-D1b* controls, and are designated as: *P<0.05; **P<0.01.

Numbers of seminal roots were equal to, or increased, for all dwarfing gene NILs compared with their respective tall controls, and consistently across both Halberd and M808S genetic backgrounds (Table 1). The GAI dwarfing gene NILs produced significantly greater numbers of seminal roots particularly in the Halberd background. Numbers of seminal roots in *Rht-B1b* or *Rht-D1b* parental NILs were generally the same as their corresponding *Rht13*-or *Rht18*-carrying NILs in the Australian varietal backgrounds. One exception was Espada where the *Rht18* NIL produced significantly fewer roots than the *Rht-D1b-*containing parent. Total root length (seminal+branch roots) (TRL) and seminal root length (seminal roots only) (SRL) were highly correlated (*r*_p_=0.93, *P*<0.01), and were equal to, or increased, in size, for all dwarfing gene NILs compared with the tall (Halberd and M808S) and most *Rht-B1b* or *Rht-D1b* (Australian varietal) controls (Table 1). The Yitpi *Rht18*-containing NIL was significantly (*P*<0.01) reduced for TRL and SRL compared with Yitpi.

Dwarfing gene NILs with increased SRL typically had greater numbers of seminal roots (e.g. Halberd containing *Rht-B1b* and *Rht18*, and M808S containing *Rht-D1b*). Numbers of seminal roots were positively correlated with increases in SRL (*r*_p_=0.63; *P*<0.01), and average SRL was consistent for all dwarfing gene NILs except reduced length for *Rht-D1c* (Halberd) and greater length for *Rht4* (M808S) and *Rht18* (Espada, Mace, and Scout). Root angle between seminal roots two and three, and seminal roots four and five, was similar for all dwarfing gene NILs, except *Rht5* which had a narrower root angle in the Halberd genetic background (Supplementary Table S1).

### Field environments

Daily maximum and minimum temperatures, rainfall, and solar radiation are given for all site×year irrigation treatments in Fig. 1. In all experiments, air temperatures were consistent with long-term minimum and maximum temperatures at each site. The Yanco in-season rainfall was 144 mm (long-term average 238 mm) while the total water supplied to the crop including irrigation was 271 mm and 363 mm for Yanco 2018 I1 (pre-anthesis irrigation only) and I2 (pre- and post-anthesis irrigation), respectively. For Condobolin 2017 I2, the in-season rainfall was 229 mm (long-term Condobolin average of 231 mm), while rainfall in Condobolin 2018 was considerably reduced, with 158 mm in the growing season with some larger rainfall events occurring later during grain filling. As irrigation was supplied at Condobolin through flood irrigation, the amount of water supplied to each sowing cannot be estimated exactly.

### Above-ground measurements under field conditions

#### Plant height

Repeatability for plant height was high (*R*^2^=0.93 ± 0.04; *P*<0.01), reflecting small dwarfing gene×environment and residual variances from the combined mixed model analysis. All dwarfing gene NILs were significantly (*P*<0.01) reduced in plant height compared with their respective tall control in Halberd, M808S, and CIMMYT genetic backgrounds (Table 2). The GAI *Rht-B1c* and *Rht-D1c*, and GAS *Rht12* dwarfing genes all produced extreme dwarf phenotypes (reductions of 42–57%), while the remaining dwarfing genes reduced plant heights by 8–39% across both Halberd and M808S backgrounds. Height reductions for the *Rht-B1b* and *Rht-D1b* GAI dwarfing genes were 8% and 13%, and 10% and 8%, respectively, in the Halberd and M808S backgrounds, and were consistent with the 10–13% height reduction for *Rht-B1b* and *Rht-D1b* genes in the CIMMYT semi-dwarf NILs (Table 2). A similar reduction in height with *Rht-B1b* and *Rht-D1b* across the Halberd, M808S, and CIMMYT genetic backgrounds reflected the statistically non-significant dwarfing gene×background interaction across all dwarfing gene NILs (data not shown). In the Australian varietal backgrounds, NILs containing the *Rht13* or *Rht18* alternative dwarfing genes were equal, or shorter, in plant height than their *Rht-B1b* or *Rht-D1b* recurrent parents (Table 2). On average, both the GAS *Rht13* and *Rht18* dwarfing genes reduced height by 9% compared with their respective *Rht-B1b*- or *Rht-D1b-*containing parents.

**Table 2.** Predicted values for growth and development characteristics for dwarfing gene near-isogenic lines (NILs) across multiple genetic backgrounds measured across multiple field environments

**Background/NIL**	**Plant height (cm)**	**Zadoks score** ^*a*^	**Anthesis biomass (kg ha** ^**–1**^)	**Grain yield (t ha** ^**–1**^)	**Harvest index**	**Maturity biomass (t ha** ^**–1**^)
*Halberd*						
HalberdT (Tall)	92	56.5	1020	3.647	0.308	11.338
Halberd *Rht-B1b*	85 (–7.6**)	55.9	1001	4.085	0.335**	12.022**
Halberd *Rht-D1b*	80 (–13.0**)	63.2**	933**	4.089**	0.350**	11.306
Halberd *Rht-B1c*	44 (–52.2**)	50.5**	906**	2.955**	0.301	8.607**
Halberd *Rht-D1c*	40 (–56.5**)	42.6**	721**	2.368**	0.157**	8.650**
Halberd *Rht5*	80 (–13.0**)	69.3**	872**	3.942	0.324**	11.880*
Halberd *Rht8*	79 (–14.1**)	68.6**	874**	3.965*	0.330**	11.843**
Halberd *Rht9*	82 (–10.9**)	69.9**	930**	3.973	0.332**	11.500
Halberd *Rht12*	53 (–42.2**)	49.1**	1021	4.261**	0.414**	9.503**
Halberd *Rht13*	83 (–9.8**)	58.6**	1023	4.142	0.323**	12.599**
Halberd *Rht18*	78 (–15.2**)	52.5**	1042	4.100*	0.334**	11.894**
*M808S*						
M808S (Tall)	112	51.1	891	2.770	0.232	12.236
M808S *Rht-B1b*	101 (–9.8**)	47.2**	895	2.445**	0.223	9.819**
M808S *Rht-D1b*	102 (–8.2**)	51.6	1099**	2.925	0.288**	9.796**
M808S *Rht-B1c*	60 (–46.6**)	42.8**	936	4.326**	0.327	12.306
M808S *Rht4*	68 (–39.3**)	58.3**	959	4.290**	0.332**	12.531
M808S *Rht5*	81 (–27.7**)	53.8*	1010*	4.202**	0.342**	11.693
M808S *Rht8*	92 (–17.9**)	56.2**	1150**	3.684**	0.322**	10.817**
*CIMMYT*						
Galvez (Tall)	108	56.7	1036	2.71**	0.246	11.130*
cv. Galvez (*Rht-B1b*)	97 (–10.2**)	58.6	1090	3.24	0.260	14.107
Nesser (Tall)	107	55.6	1218	2.84**	0.231**	12.403
cv. Nesser (*Rht-B1b*)	93 (–13.1**)	56.4	1135	4.19	0.358	11.408
Pavon (Tall)	105	57.1	967*	2.50	0.184**	14.788*
cv. Pavon (*Rht-D1b*)	94 (–10.5**)	57.1	1199	2.87	0.273	10.640
Seri (Tall)	101	60.7**	847*	3.83*	0.320*	11.472
cv. Seri (*Rht-B1b*)	88 (–12.1**)	55.3	1058	4.41	0.393	11.362
*Australian varietal backgrounds*						
cv. Espada (*Rht-D1b*)	84	59.5	1379	4.602	0.343	12.506
Espada *Rht13*	77 (–8.3**)	63.8**	860**	4.096*	0.312**	13.981*
Espada *Rht18*	83 (–1.2)	52.2*	1061**	4.348	0.336	12.657
cv. Gregory (*Rht-B1b*)	99	49.3	1138	4.061	0.325	12.228
Gregory *Rht13*	85 (–14.1**)	45.8*	1058*	3.448**	0.244**	12.375
Gregory *Rht18*	88 (–11.1**)	53.5**	1242*	4.014	0.310	12.778
cv. Mace (*Rht-D1b*)	89	57.4	1155	4.688	0.335	14.528
Mace *Rht18*	87 (–2.2)	55.6	1172	4.769	0.366**	13.055**
cv. Magenta (*Rht-D1b*)	82	49.0	1250	4.162	0.310	12.686
Magenta *Rht18*	89 (+8.5**)	48.3	993**	3.541**	0.285	11.502
cv. Scout (*Rht-D1b*)	87	58.7	1066	4.175	0.307	14.629
Scout *Rht18*	75 (–13.8**)	68.3**	956**	4.517	0.324	11.499**
cv. Yitpi (*Rht-D1b*)	90	51.8	997	4.175	0.307	12.457
Yitpi *Rht18*	83 (–7.8**)	55.5**	1020	4.640*	0.363**	11.338

*Rht*, reduced major height gene with *Rht-B1b*, *Rht-B1c*, *Rht-D1b*, and *Rht-D1c* genes conferring GA insensitivity (GAI), and *Rht4*, *Rht5*, *Rht8, Rht9*, *Rht12*, *Rht13*, and *Rht18* genes conferring GA sensitivity (GAS). Zadok’s scores were undertaken 115–118 d after sowing. Percentage reduction in plant height relative to control is given in parentheses. Significance levels represent comparisons with the tall or *Rht-B1b*, *Rht-D1b* controls, and are designated as: *P<0.05; **P<0.01.

^*a*^ Measured at Yanco in 2018 only.

#### Development

Development (as Zadoks score) varied between NILs in all genetic backgrounds. Generally, NILs containing alternative dwarfing genes *Rht4*, *Rht5*, *Rht8*, and *Rht9* accelerated development toward anthesis, whereas *Rht-B1c*, *Rht-D1c*, and *Rht12* slowed development when compared with their respective Halberd tall and M808S controls (Table 2). The dwarfing gene×genetic background interaction was statistically significant, with the *Rht-B1b* gene delaying development relative to the tall control in the M808S and Seri genetic backgrounds (*P*<0.01), but not in the Halberd background. Conversely, the *Rht-D1b* gene hastened development in the Halberd background (*P*<0.01) but was not statistically different from tall controls in the M808S and CIMMYT genetic backgrounds (Table 2). In the Halberd background, the *Rht13* gene hastened, and *Rht18* delayed, development. Development was generally accelerated and anthesis date earlier with *Rht13* and *Rht18* compared with *Rht-B1b* or *Rht-D1b* in the Australian varietal backgrounds. Exceptions were the Espada and Gregory backgrounds where *Rht13* and *Rht18* genes varied in their effect on plant development.

#### Crop biomass and grain yield

Grain yields for the majority of dwarfing gene NILs were similar to, or greater than, those of their respective tall controls (Table 2). The dwarfing gene×genetic background interaction was statistically significant, with some dwarfing gene NILs varying for grain yield across genetic backgrounds. For example, grain yield for the extreme dwarfing gene *Rht-B1c* NIL was significantly less than for the tall Halberd control but significantly greater than for the M808S tall control. The *Rht-D1b*-carrying NILs were significantly higher yielding in the Halberd and most CIMMYT backgrounds but not in M808S, while *Rht-B1b* NILs were lower yielding in the M808S background but not in Halberd or Seri backgrounds (Table 2). Despite the change in ranking for *Rht-B1b*, *Rht-B1c*, and *Rht-D1b*, some genes (e.g. *Rht8*) were consistent for grain yield across genetic backgrounds. Grain yields for the *Rht18* NILs were commonly equal to, or greater than, yields for their tall or *Rht-B1b-* or *Rht-D1b-*carrying parents. In contrast, *Rht13* was associated with significantly reduced grain yield in the Espada and Gregory backgrounds (Table 2).

Across all genetic backgrounds, increases in HI were positively correlated with changes in grain yield (*r*_p_=0.84, *P*<0.01). Dwarfing gene×genetic background interaction was non-significant, with HI being commonly greater for dwarfing gene NILs except for *Rht-B1c* and *Rht-B1b* in the Halberd and M808S backgrounds, respectively (Table 2). In the Australian varietal backgrounds, *Rht13* was associated with significantly reduced HI whereas *Rht18* was commonly equal to or greater for HI (Table 2).

Differences among dwarfing gene-carrying NILs were large and statistically significant for anthesis and maturity biomass (Table 2). The dwarfing gene×genetic background interaction was statistically significant with *Rht-D1b*-, *Rht5*-, and *Rht8*-containing NILs, significantly smaller for anthesis biomass than the tall control in the Halberd background, but larger than M808S in the M808S background. Extreme dwarf GAI *Rht-B1c* or *Rht-D1c* NILs produced among the smallest anthesis biomass in the Halberd background, whereas the GAS *Rht12* extreme dwarf NIL was not statistically different from the tall control. A large dwarfing gene×background interaction for maturity biomass was strongly evident with reranking of means for the GAI *Rht-B1b*- or *Rht-D1b-*containing NILs, and GAS *Rht5* and *Rht8* dwarfing gene NILs between the Halberd and M808S backgrounds (Table 2). The *Rht13* dwarfing gene NILs were equal to or greater in maturity biomass across all backgrounds, while *Rht18* NILs were larger in biomass in the Halberd background and equal to, or smaller than, their *Rht-B1b* or *Rht-D1b* parents in the Australian varietal backgrounds.

### Phenotypic variation for root depth under field conditions

In the full mixed linear model, dwarfing gene NIL, genetic background, operator (i.e. root counter), and length of the soil core were all statistically significant (*P*<0.01) for MRD and RPR. Repeatability for MRD was intermediate in size (*R*^2^=0.56 ± 0.09; *P*<0.01), reflecting a small and non-significant dwarfing gene×environment interaction but large residual and sampling variances (data not shown). Repeatability for RPR was also intermediate in size (*R*^2^=0.52 ± 0.10; *P*<0.01). As for MRD, the dwarfing gene×environment interaction was small and not significant, while residual and sampling variances were large (data not shown).

#### Maximum rooting depth

**Fig. 2.**
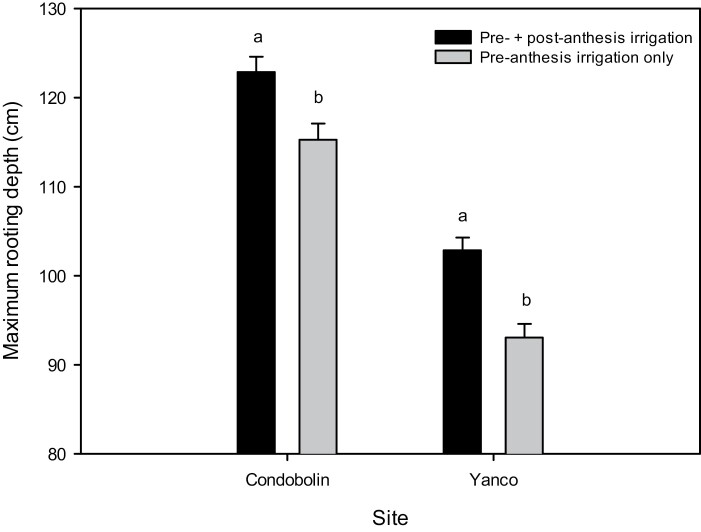
Mean maximum rooting depth (and SEs) for pre- and pre- and post-anthesis irrigated plots at Condobolin and Yanco in 2018. Different letters within each site denote statistically significant (*P*<0.05) differences for maximum rooting depth with different irrigation regimes.

Maximum rooting depth varied significantly (*P*<0.01) across experiments, ranging from 133 cm at Condobolin in 2017 (with post-anthesis irrigation, I2) to 93 cm at Yanco in 2018 (pre-anthesis irrigation only, I1). In 2018, mean MRD for pre- and pre-+post-anthesis irrigation was 115 cm and 123 cm (Condobolin), and 93 cm and 103 cm (Yanco), respectively (Fig. 2). The increase in MRD with post-anthesis irrigation was statistically significant (*P*<0.01) at 6.9% and 9.9% for Condobolin and Yanco, respectively, and was consistent across the two sites with their contrasting soil types.

The MRD was generally equal to or greater at maturity for dwarfing gene NILs when compared with their respective tall or *Rht-B1b/Rht-D1b* controls across genetic backgrounds (Table 3). Background genetic effects were small but significant (*P*<0.01), with the greatest MRD in the Australian varietal (109 mm) and smallest in the CIMMYT (99 mm) genetic backgrounds (data not shown). The background×dwarfing gene interaction was small and not statistically significant, with NILs ranking similarly across backgrounds for MRD. One exception was the extreme GAI *Rht-B1c*-carrying NIL which was similar in MRD to the Halberd control (+6%) but significantly deeper (+23%) than M808S (Table 3). The MRD of *Rht8* NILs was significantly deeper in both Halberd and M808S genetic backgrounds (+5%). The *Rht-B1b* or *Rht-D1b* NILs were not statistically different for MRD from the tall controls in the Halberd, M808S, and three of the four CIMMYT genetic backgrounds (Table 3). The *Rht18* NIL varied in MRD across Australian varietal backgrounds (+6% to 24%), and were on average significantly (*P*<0.01) deeper rooting (+16%) than their *Rht-B1b* or *Rht-D1b* parents (Table 3). To a lesser extent, the *Rht13* dwarfing gene averaged +7% greater rooting depth over commercial parents.

**Table 3.** Predicted values for maximum root depth and root penetration rate for dwarfing gene near-isogenic lines (NILs) across multiple genetic backgrounds measured across multiple field environments

**Background/NIL**	**Maximum rooting depth (cm)**	**Root penetration rate (mm d** ^**–1**^)^*a*^
*Halberd*		
HalberdT (Tall)	110	8.15
Halberd *Rht-B1b*	113 (+2.2)	8.27 (+1.9)
Halberd *Rht-D1b*	110 (+0.1)	8.52 (+4.5)
Halberd *Rht-B1c*	112 (+1.8)	8.47 (+4.0)
Halberd *Rht-D1c*	102 (–7.9**)	7.22 (–11.9*)
Halberd *Rht5*	110 (–0.3)	8.63 (+6.0*)
Halberd *Rht8*	113 (+2.6*)	8.96 (+9.9**)
Halberd *Rht9*	115 (+4.3*)	8.97 (+10.1**)
Halberd *Rht12*	116 (+5.2**)	8.99 (+10.4**)
Halberd *Rht13*	114 (+3.8*)	8.66 (+6.3**)
Halberd *Rht18*	113 (+2.5*)	8.22 (+0.8)
*M808S*		
M808S (Tall)	106	7.62
M808S *Rht-B1b*	107 (+0.4)	7.63 (+0.1)
M808S *Rht-D1b*	112 (+5.2)	8.23 (+8.0)
M808S *Rht-B1c*	133 (+25.2**)	9.56 (+25.5**)
M808S *Rht4*	116 (+9.4*)	8.91 (16.9*)
M808S *Rht5*	112 (+4.8)	8.19 (+7.5)
M808S *Rht8*	119 (+12.5*)	9.08 (+19.2**)
*CIMMYT*		
Galvez (Tall)	116	8.71
cv. Galvez (*Rht-B1b*)	118 (+1.3)	8.71 (+0.0)
Nesser (Tall)	98	7.38
cv. Nesser (*Rht-B1b*)	109 (+9.7)	8.10 (+9.9)
Pavon (Tall)	110	8.24
cv. Pavon (*Rht-D1b*)	107 (–2.7)	8.08 (-1.9)
Seri (Tall)	98	7.48
cv. Seri (*Rht-B1b*)	118 (+20.2**)	8.38 (+12.5)
*Australian varietal backgrounds*		
cv. Espada (*Rht-D1b*)	105	7.69
Espada *Rht13*	110 (+4.6)	8.53 (+10.4)
Espada *Rht18*	127 (+21.4**)	9.59 (+24.6**)
cv. Gregory (*Rht-B1b*)	116	8.38
Gregory *Rht13*	122 (+5.9)	8.81 (+5.2)
Gregory *Rht18*	121 (+4.7)	9.08 (+8.4)
cv. Mace (*Rht-D1b*)	111	8.11
Mace *Rht18*	122 (+9.7**)	9.39 (+15.7*)
cv. Magenta (*Rht-D1b*)	115	8.30
Magenta *Rht18*	129 (+12.2*)	9.27 (+11.7*)
cv. Scout (*Rht-D1b*)	111	8.41
Scout *Rht18*	121 (+8.6*)	9.66 (+14.8*)
cv. Yitpi (*Rht-D1b*)	111	8.08
Yitpi *Rht18*	130 (+17.6**)	10.4 (+28.7**)

*Rht*, reduced major height gene with *Rht-B1b*, *Rht-B1c*, *Rht-D1b*, and *Rht-D1c* genes conferring GA insensitivity (GAI), and *Rht4*, *Rht5*, *Rht8, Rht9*, *Rht12*, *Rht13*, and *Rht18* genes conferring GA sensitivity (GAS). Zadok’s scores were undertaken 115–118 d after sowing. Percentage reduction in plant height relative to control is given in parentheses. Significance levels represent comparisons with the tall or *Rht-B1b*, *Rht-D1b* controls, and are designated as: *P<0.05; **P<0.01.

^*a*^ Measured at Yanco in 2018 only.

**Fig. 3.**
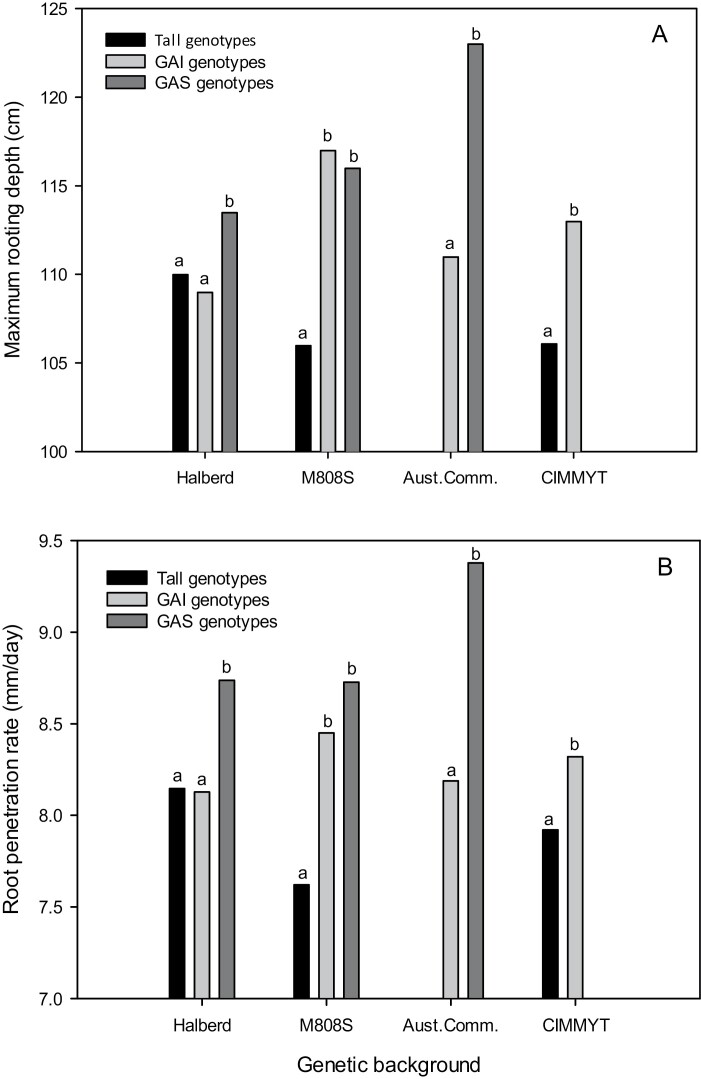
Dwarfing gene class means for (A) maximum rooting depth and (B) root penetration rate across each of the four tested genotype backgrounds; gibberellic acid insensitive (GAI), gibberellic acid sensitive (GAS), and Tall (lacking any dwarfing genes). Dwarfing genes are provided for each class in the text. Different letters within a genetic background denote statistically significant (*P*<0.05) differences for dwarfing gene classes.

Figure 3 summarizes mean MRD for all NILs carrying GAI, GAS, or no (tall) dwarfing genes in the four background sets. Both GAS- and GAI-containing dwarfing gene NILs were significantly deeper rooting than the tall controls in the Halberd and M808S backgrounds, and not different from each other in the M808S background. However, in both the Halberd and Australian variety backgrounds, GAS NILs were significantly (*P*<0.01) greater than GAI NILs for MRD (Fig. 3).

#### Root penetration rate

RPR was only measured at Yanco in 2018. The average RPR was significantly (*P*<0.01) greater (+13%) in the post-anthesis irrigation treatment (8.94 mm d^–1^) compared with the pre-anthesis irrigation treatment (8.15 mm d^–1^).

Genetic background differences were large and significant (*P*<0.01) for RPR, ranging from 8.2 mm d^–1^ for NILs in the CIMMYT background, to intermediate 8.4 mm d^–1^ and 8.5 mm d^–1^ in the M808S and Halberd backgrounds, respectively, and a high rate of 8.8 mm d^–1^ for NILs in Australian varietal backgrounds (Fig. 3). The dwarfing gene×background interaction was small but statistically significant. The GAI NILs were, on average, larger than the tall NILs for RPR in the M808S and CIMMYT backgrounds, but this largely reflected the greater RPR for *Rht-B1c* and Seri *Rht-B1b* in the M808S and CIMMYT backgrounds, respectively (Table 3). The RPR was significantly (*P*<0.01) greater for GAS than GAI dwarfing gene NILs in the Halberd and Australian varietal backgrounds, and were larger but not significantly different in the M808S background (Fig. 3). The *Rht8* dwarfing NIL was significantly (*P*<0.01) larger for RPR in the Halberd and M808S backgrounds, and *Rht9* and *Rht12* dwarfing NILs were significantly greater in the Halberd background (Table 3). The *Rht13*-containing NILs were associated with significantly (*P*<0.01) increased RPR in the Halberd background but were not different from *Rht-B1b* or *D1b* NILs in the Australian varietal backgrounds (Table 3). In contrast, the *Rht18* NILs were equal to or significantly greater (*P*<0.01) for RPR in Australian varietal backgrounds.

### Genotypic relationships for root and agronomic phenotypes

Across both irrigation regimes at Yanco in 2018, genotypic increases in MRD were moderately correlated with increases in RPR (*r*_g_=0.46 ± 0.06; *P*<0.01). Delayed development (i.e. lower Zadoks scores) were moderately correlated with genotypic reductions in RPR (*r*_g_=0.36 ± 0.09; *P*<0.01) despite genotypic increases in MRD with later flowering (*r*_g_= –0.38 ± 0.08; *P*<0.05) (Table 4).

**Table 4.** Genetic correlations (±SE) for maximum root depth and root penetration rates with agronomic traits assessed at Yanco in 2018 for both irrigated and rainfed environments

Character	Maximum rooting depth	Root penetration rate
Zadoks score	–0.38 ± 0.08**	0.36 ± 0.09**
Mature plant height	–0.16 ± 0.11ns	–0.03 ± 0.16ns
Anthesis biomass	0.09 ± 0.10ns	–0.03 ± 0.23ns
Grain yield (all)^*a*^	0.45 ± 0.16**	0.53 ± 0.10**
- Irrigated only	0.35 ± 0.14*	0.33 ± 0.15*
- Droughted only	0.57 ± 0.18**	0.75 ± 0.10**
Harvest index (all)^*a*^	0.56 ± 0.14**	0.47 ± 0.22*
- Irrigated only	0.49 ± 0.16**	0.36 ± 0.15*
- Droughted only	0.58 ± 0.18**	0.76 ± 0.08**
Maturity biomass (all)^*a*^	0.20 ± 0.24ns	0.21 ± 0.14ns
- Irrigated only	0.30 ± 0.21ns	0.13 ± 0.15ns
- Droughted only	0.06 ± 0.24ns	0.22 ± 0.18ns

Significance levels are designated as: *P< .05; **P< 0.01; and ns, *P*>0.05.

^*a*^ Combined irrigated and droughted environments.

Genotypic increases in MRD were positively correlated with increases in grain yield and HI (*P*<0.01), and were independent of changes in plant height, development, and anthesis and maturity biomass (Table 4). Similarly, genotypic increases in RPR were positively correlated with increases in grain yield and HI (*P*<0.01), and lower, albeit negatively correlated with Zadoks score. Plant height and both anthesis and maturity biomass were genetically uncorrelated with RPR. Genetic correlations were estimated for grain yield, HI, and maturity biomass separately for irrigated and rainfed environments (Table 4). The genetic correlations for MRD and RPR with both grain yield and HI were strongest under droughted conditions, and not significantly different from zero for maturity biomass. The genetic correlation for yield was particularly strong and positive with RPR, and consistent with greater HI but not biomass at maturity.

## Discussion

### Influence of dwarfing genes on early growth under controlled conditions

Seeds used in the controlled-environment study were carefully standardized to: (i) be the same weight; and (ii) sourced from the same glasshouse growing environment to minimize confounding dwarfing gene and maternal differences for germination and early shoot/root growth (Rebetzke *et al.*, 2022). Up to 80% of the variation in seedling biomass can be directly attributed to differences in seed source and size due to maternal environment differences (Roach and Wulff, 1987). After selecting seeds for uniformity of weight, small genetic differences in early seedling growth including the influence of different dwarfing genes would be undetected or even biased if these important covariances were ignored.

The shorter lengths of leaf one in the controlled-environment study (Table 1) confirmed previously observed reductions in seedling growth with the GAI dwarfing genes *Rht-B1b*, *Rht-D1b*, *Rht-B1c*, and *Rht-D1c* (e.g. Botwright *et al*., 2001, 2005; Addisu *et al.*, 2009; Rebetzke *et al.*, 2014). Further, the greater leaf length of GAS *Rht5*, *Rht8*, *Rht9*, *Rht12*, *Rht13*, and *Rht18* dwarfing genes was consistent with greater leaf size reported for these genes elsewhere (e.g. Addisu *et al.*, 2009; Rebetzke *et al.*, 2012).

The numbers of seminal roots varied together with SRL (*r*_p_=0.63, *P*<0.01), highlighting the importance of factors contributing to greater seminal root number in seedling root architecture. Seminal root number is under strong maternal and genetic control (Meyer, 1976, and references therein), and increases in embryo size are linked to increased frequency and size of seminal roots four, five, and six (Rebetzke *et al.*, 2022). The larger number of seminal roots in NILs containing GAI *Rht-B1_* and *Rht-D1_* alleles contributed to their greater SRL, while average SRL was not different from that of tall controls (except *Rht-D1c* which produced significantly shorter seminal roots). The reduced average SRL for *Rht-D1c* seedlings is consistent with other reports of decreased TRL of *Rht-D1c*-containing NIL seedlings in controlled environments (Wojciechowski *et al.*, 2009; Bai *et al.*, 2013).

Narrow seminal root angles contribute to greater rooting depth in rice (Oyanagi *et al.*, 1993) and wheat (Richard *et al.*, 2015). In the present study, only the single *Rht5*-carrying Halberd NIL produced a significantly narrower root angle (seminal roots four and five) than the respective tall control (Table 1). Root angle for the *Rht5* NIL in the M808S background was not different from that of the tall control M808S. Neither the Halberd nor M808S *Rht5* NILs were significantly different in MRD from their respective tall controls in the field (Table 3). Root angle measured in growth cabinet-grown seedlings was unrelated to root angle measured under field conditions (Rich *et al.*, 2020). Therefore, a potential effect of narrower root angles on MRD for specific genotypes cannot be dismissed solely based on our seedling studies.

### Genotypic variation in plant height under field conditions

All dwarfing gene NILs reduced mature plant height. Height reductions varied from 10% (*Rht-B1b*) to 55% (*Rht-D1c*), and were consistent in effect across contrasting genetic backgrounds (Table 2). The NILs carrying the *Rht-B1c*, *Rht-D1c*, and *Rht12* dwarfing genes exhibited the largest reductions in plant height, and *Rht-B1b* and *Rht-D1b* NILs the smallest height reduction. The change in plant height was consistent with ranking for *Rht-B1b*, *Rht-D1b*, *Rht-B1c*, *Rht8*, *Rht-D1c*, and *Rht12* dwarfing gene NILs in the winter varietal background Mercia (Addisu *et al.*, 2009). However, whereas the *Rht8* NIL was similar in height to *Rht-B1b* and *Rht-D1b* NILs in the winter Mercia (Addisu *et al.*, 2009) and Paragon (Casebow *et al.*, 2016) backgrounds, height reduction was greater for *Rht8* NILs in the spring Halberd and M808S backgrounds.

Australian varieties Espada and Gregory were both represented with GAS *Rht13* and *Rht18* dwarfing gene NILs, with *Rht13* reducing height to a greater extent than *Rht18*. A strong height-reducing effect for *Rht13* was also reported by Divashuk *et al.* (2020) and Rebetzke *et al.* (2011). Notwithstanding, the extent of height reduction with *Rht13* was contingent on genetic background (cf. Halberd, Espada, and Gregory backgrounds) (Table 2). Similarly, *Rht18* NILs varied in height depending on genetic background (cf. Mace and Scout backgrounds). The potential for differential ranking for plant height across dwarfing genes emphasizes the need for assessment across both genetic backgrounds and environments.

The large number of random lines representing each dwarfing gene in our study, together with the consistency in ranking of height across the different genetic backgrounds (Table 1) and dwarfing genes reported elsewhere (e.g. Addisu *et al.*, 2009), increases confidence in the genetic effects of dwarfing genes on root architecture and other measured traits.

### Root depth varies across dwarfing genes

Genotypic variation for MRD and RPR was large and repeatable within and across the different genetic backgrounds. Repeatability was not as large as for plant height (cf. 0.92, 0.56, and 0.52 for height, MRD, and RPR, respectively), but was moderately consistent with heritabilities for MRD in Wasson *et al.* (2017) and larger than heritabilities reported elsewhere (e.g. Guo *et al.*, 2020). Spatial variation within and between field plots together with factors including coring depth and root count can all contribute to the large sampling variation commonly encountered with field root phenotyping (Wasson *et al.*, 2012; Guo *et al.*, 2020). As reported in Guo *et al.* (2020), genotype (here dwarfing gene×genetic background) variances for MRD and RPR were large relative to genotype×environment interaction variances, suggesting that only a few well-managed environments containing deep and unconstrained soils are needed for assessing genotypic differences in MRD and RPR.

Time of anthesis varied across dwarfing genes and backgrounds (Table 2). Anthesis in *Rht-B1b*-, *Rht-D1b*-, *Rht13*-, and *Rht18*-carrying NILs was delayed in some but not all backgrounds, whereas *Rht-B1c*-containing NILs were consistently delayed and *Rht8* NILs were consistently quicker to reach anthesis. Dwarfing genes have been reported to vary in their influence on plant development (e.g. Addisu *et al.*, 2010; Rebetzke *et al.*, 2012). The *Rht8* gene is associated with earlier flowering through genetic linkage with the photoperiod insensitivity gene *Ppd-D1a* on chromosone 2DS (Worland *et al.*, 1998). Delays in flowering extend the period for root growth to increase MRD (Kirkegaard and Lilley, 2007). In the present study, the range in development across dwarfing gene NILs was considered small and the genetic correlation between Zadoks score and MRD modest in size. Further, in the controlled-environment study, genotypic differences for root traits were apparent on seedlings assessed at the same early growth and development stage. Together, these results suggest that dwarfing genes influenced root growth independent of development in the NILs studied.

Delayed flowering across genotypes was modestly correlated with genotypic increases in MRD consistent with reports of extended root growth with slower development to flowering (Kirkegaard and Lilley, 2007). Delaying flowering should allow for deeper root growth and access to available water, but later flowering in many environments can delay grain growth into hot and dry conditions to reduce grain yield and quality. Genotypic variation in RPR was large and repeatable, and genetically correlated with MRD, whereas development score was negatively correlated with RPR. Faster rates of root growth may provide a robust and inexpensive surrogate for MRD independent of crop development. Potential differences in RPR could be simply assessed for large numbers of genotypes in tubes containing sieved field or commercial soils in controlled environments. Further studies are needed to validate the potential for high-throughput phenotyping of RPR in controlled and field environments.

Maximum root depth and RPR were significantly greater for most GAS and GAI dwarfing gene-containing NILs when compared with the respective controls (Fig. 3). Differences between the GAS and GAI groups were in themselves smaller but statistically significant, with the GAS dwarfing NILs an average 2.1% and 4.6% greater for MRD and RPR, respectively. Our data highlight the opportunity for use of GAS dwarfing genes to improve rooting depth, and the consistency of GAS dwarfing NILs for MRD and RPR across multiple genetic backgrounds. For example, MRD in *Rht13*- and *Rht18*-containing NILs was equal to, or greater than, the respective *Rht-B1b*- or *Rht-D1b*-containing parents in the Australian varietal backgrounds. The advantages of increased coleoptile length and early shoot vigour with GAS dwarfing genes is widely accepted (e.g. Rebetzke *et al.*, 2012; Zhao *et al.*, 2022), and *Rht18*-based, semi-dwarf wheat varieties are now available commercially. However, the influence of GAS dwarfing genes on root growth and architecture remains to be understood. Even small increases in MRD can increase late-season access to water deep in the soil profile to improve the marginal water use efficiency of rainfed crops (Kirkegaard *et al.*, 2007). The value of deep soil water was demonstrated in the experiment at Yanco in 2018 with the strong and positive genetic correlation for grain yield and both MRD and RPR, and particularly in the droughted environment (Table 3). Further, the strong genetic correlation for root depth parameters with HI but not biomass at maturity suggests that genotypic differences in post-anthesis water use may be contributing to increases in grain yield. Greater water use after flowering has previously been shown to be associated with increases in HI and grain yield in droughted environments (Passioura, 1977), while deeper roots have been shown to increase water use and wheat yields across different drought experiments (e.g. Palta *et al.*, 2011;El Hassouni *et al*., 2018; Li *et al.*, 2019).

### Improving inference when phenotyping root growth and architecture

The need for multiple, deep soil cores to account for root plasticity with soil variability has limited most genetic studies to seedling growth in controlled environments or early growth stages in the field (e.g. Wojciechowski *et al.*, 2009). However, poor correlation between assessments representing different stages of crop development, growth cabinet and field, and different soil mimics (e.g. gels and hydroponics) (Wojciechowski *et al.*, 2009) highlight uncertainty when concluding among studies about genetic factors affecting root architecture.

The ranking of genotypes for seedling root architecture correlates poorly with root architecture in field-grown seedlings (Wojciechowski *et al.*, 2009) and adult (Rich *et al.*, 2020) plants. Seed source and size are well known to affect seedling shoot and root growth (Roach and Wulff, 1987), yet few seedling studies acknowledge the importance of controlling maternal factors when comparing among genotypes. Indeed, seed size is rarely reported or standardized, and dwarfing genes are well established to influence seed number and seed size (e.g. Flintham *et al.*, 1997; Butler *et al.*, 2005). Bai *et al.* (2013) reported strong positive correlations for seed weight and plant height, with a range of seedling root size attributes measured on genotypes in a wheat mapping population. Further, their quantitative trait locus (QTL) analysis identified overlapping genomic regions for seed weight, seedling root growth, and plant height (including the *Rht-D1* locus on chromosome 4D). Their results support other research (e.g. Singh *et al.*, 2017) highlighting the need to control seed weight in seedling root and shoot assessment, and particularly for factors known to affect seed weight such as plant height. Covariance analysis has been used to adjust for large mean seed size differences between dwarfing gene NILs (e.g. Wojciechowski *et al.*, 2009). However, as plant height and seed size differences are confounded (i.e. not independent), inference here on the effects of dwarfing genes on seedling root growth should be treated with caution.

Comparisons of root architecture between short and tall height phenotypes have rarely been undertaken on near isolines or siblings to control genetic background and restrict inference to specific dwarfing genes. For example, numerous studies report reductions in sizes of root systems of modern *Rht-B1b* and *Rht-D1b* semi-dwarf varieties in comparisons between modern and older, tall or landrace wheat varieties (e.g. Waines and Ehdaie, 2007; Bai *et al.*, 2013; Bektas *et al.*, 2016; Subira *et al.*, 2016; Aziz *et al.*, 2017; Friedli *et al.*, 2019; Fradgley *et al.*, 2020). Indeed, comparisons of field-grown tall and GAI semi-dwarf NILs (at flowering) were not different for root length or biomass (McCaig and Morgan, 1993), or semi-dwarf NILs were larger for root biomass and not length (Miralles *et al.*, 1997).

### Evidence for post-anthesis root growth?

Roots were measured growing an average 6.9–9.9% deeper for plots supplied with an additional 1–2 irrigations at and/or after anthesis. The increased MRD was consistent across the two sites despite their contrasting in soil type from a light clay–loam to a heavier vertosol. This evidence for post-anthesis root growth is counter to the belief that root growth ceases at anthesis (Siddique *et al.*, 1990), and that assimilates in demand for grain growth are preferentially allocated away from growing roots (Andersson *et al.*, 2005; Kirkegaard and Lilley, 2007). Additional root growth at depth was unlikely to be due to deeper wetting with irrigation to depths below 90 cm (Asseng *et al.*, 1998), suggesting continued partitioning of assimilate to roots. Post-anthesis root growth has been reported for different wheat varieties assessed in root boxes in a controlled environment (Manschadi *et al.*, 2006), and with post-anthesis fungicide application in the field (Ford *et al.*, 2006). In the latter, increases in root length of 45.6% and 11.5% were measured in fungicide-treated plots in two seasons, with the largest increase in the season with the greatest fungal infection. Similarly, Gregory *et al.* (1978) reported post-anthesis wheat root growth below 80 cm depth despite a reduction in overall root dry weight. They hypothesized that senescence in some parts of the root system enabled continued root growth and exploration at depth. These different findings suggest that root growth may continue at depth if conditions are favourable (i.e. soils contain adequate moisture and are non-toxic), and support the need for root phenotyping to be undertaken at maturity.

### Conclusions

This is the first report of field assessment of wheat dwarfing gene NILs for MRD and RPR, and their relationship with grain yield across multiple genetic backgrounds. All dwarfing genes decreased mature plant height and, in most cases, were equal to or greater for MRD and RPR compared with their respective tall or GAS dwarfing gene controls. Deeper roots were genetically associated with greater HI and increased grain yield particularly in droughted environments. The GAS dwarfing genes showed promise in increasing root depth while maintaining reductions in plant height. Our results quantified the effects of the different dwarfing genes on root growth to aid in informing breeders when selecting genotypes with improved water and nutrient uptake for droughted environments.

## Supplementary data

The following supplementary data are available at *JXB* online.

Table S1. Mean values for different root architecture traits measured on near-isogenic wheat genotypes in a root pouch screen in a controlled environment.

erac306_suppl_Supplementary_Material

## Data Availability

The data supporting the findings of this study are available from the corresponding author, Greg Rebetzke, upon request.

## Abbreviations

GA: gibberellic acid
GAI: gibberellic acid insensitive
GAS: gibberellic acid sensitive
HI: harvest index
I1: irrigation regime 1
I2: irrigation regime 2
MRD: maximum root depth
NIL: near isogenic line
Rht: reduced height
RPR: root penetration rate
TRL: total root length
SRL: total seminal root length
